# Controlling Oxygen Mobility in Ruddlesden–Popper Oxides

**DOI:** 10.3390/ma10040368

**Published:** 2017-03-31

**Authors:** Dongkyu Lee, Ho Nyung Lee

**Affiliations:** Oak Ridge National Laboratory, Oak Ridge, TN 37831, USA; dklee97@gmail.com

**Keywords:** ruddlesden-popper oxides, perovskite oxides, layered perovskite oxides, mixed ionic and electronic conductors, oxygen ion migration, oxygen diffusion, anisotropy, solid oxide fuel cells, thermal expansion coefficients, chemical expansion

## Abstract

Discovering new energy materials is a key step toward satisfying the needs for next-generation energy conversion and storage devices. Among the various types of oxides, Ruddlesden–Popper (RP) oxides (A_2_BO_4_) are promising candidates for electrochemical energy devices, such as solid oxide fuel cells, owing to their attractive physicochemical properties, including the anisotropic nature of oxygen migration and controllable stoichiometry from oxygen excess to oxygen deficiency. Thus, understanding and controlling the kinetics of oxygen transport are essential for designing optimized materials to use in electrochemical energy devices. In this review, we first discuss the basic mechanisms of oxygen migration in RP oxides depending on oxygen nonstoichiometry. We then focus on the effect of changes in the defect concentration, crystallographic orientation, and strain on the oxygen migration in RP oxides. We also briefly review their thermal and chemical stability. Finally, we conclude with a perspective on potential research directions for future investigation to facilitate controlling oxygen ion migration in RP oxides.

## 1. Introduction

To meet the increasing demand for electrochemical energy conversion and storage devices, including solid oxide fuel cells (SOFCs) and oxygen membranes, the development of new oxide materials is a critical element. So far, substantial efforts have been focused on developing ABO_3_ perovskite oxides, which exhibit fascinating physicochemical properties—i.e., high electronic and ionic conductivities and high catalytic activities [1,2,3]—for use in energy applications. Mixed ionic and electronic conductors (MIECs), such as La_1−x_Sr_x_CoO_3−δ_ (LSC_113_) [4,5,6,7,8,9,10,11,12] and La_1−x_Sr_x_Co_1−y_Fe_y_O_3−δ_ (LSCF_113_) [13,14,15,16,17,18,19,20,21,22,23,24,25,26], that show high degrees of oxygen deficiency are commonly used to promote oxygen diffusivity and surface exchange kinetics at intermediate temperatures. However, these materials suffer from some serious inherent limitations, including poor thermal and chemical stability [27,28,29,30] and long-term instability [22,31,32,33,34].

**Figure 1:**
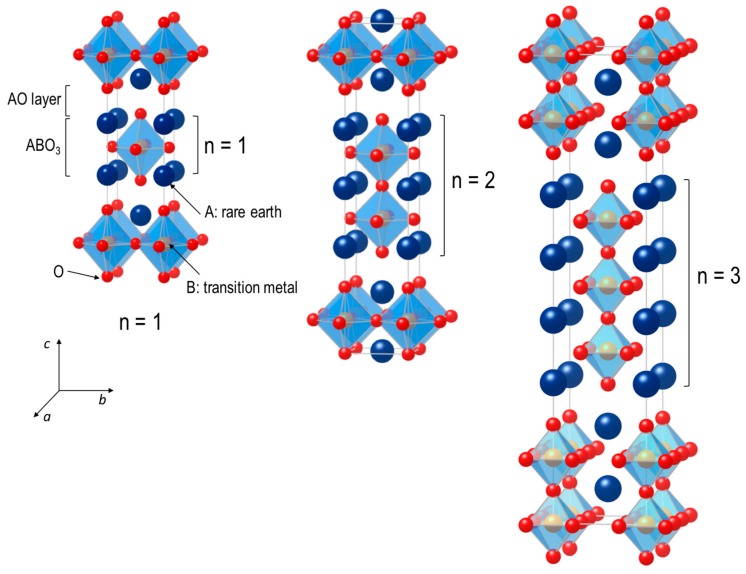
Schematic crystal structures of *n* = 1, 2 and 3 members of Ruddlesden–Popper type A_n+1_B_n_O_3n+1_ are shown. The denotation of *n* represents the number of stacked octahedral layers separated by a rock salt AO layer.

Ruddlesden–Popper (RP) oxides have shown very attractive and versatile physical properties such as superconductivity [35,36,37,38], magnetoresistance [39,40,41], and mixed ionic and electronic conductivity [42,43,44,45,46], which are beneficial for many energy and electronic devices. The general formula of RP phases can be written as A_n+1_B_n_O_3n+1_ (*n* ≥ 1) [47]. The RP phases comprise *n* consecutive perovskite layers (ABO_3_) alternating with rock-salt layers (AO) along the crystallographic *c*-axis direction. Their formula can be represented by (AO)**·**(ABO_3_)*_n_*, where *n* represents the number of connected layers of vertex-sharing BO_6_ octahedra [48]. Figure 1 presents the ideal tetragonal unit-cells for *n* = 1, 2, and 3, which correspond to the stoichiometric compounds with the same space group, *I4/mmm*. For *n* > 1, the additional ABO_3_ blocks are introduced between two AO rock-salt layers. Commonly, these materials consist of rare or alkaline earth A-site cations with transition metals on the B site, forming an extensive series of compositions. The A-site cations have a coordination number of 9, locating at the boundary between the two types of layers, while the B-site cations are positioned at the center of an octahedron formed by six oxygen anions. Similar to ABO_3_ perovskite oxides, the RP phases show a rather high structural flexibility in the oxygen stoichiometry.

Of particular interest in the case of electrochemical energy applications such as SOFCs is the A_2_BO_4_ system (*n* = 1) because their oxygen surface exchange kinetics and oxygen diffusion are higher than those in ABO_3_ oxides [42,49,50,51,52,53]. Owing to the adjustment of two different structural units, i.e., ABO_3_ and AO, within the lattice, the A_2_BO_4_ structure exhibits strong anisotropic features. As described above, the B-site cations are coordinated by six oxygen anions, but the B-O bond lengths are different because of the Jahn–Teller effect caused by the valence state of the B-site cations [54]. This results in two types of oxygen species in the BO_6_ octahedra, which are referred to as “apical” and “equatorial” oxygen. In the A_2_BO_4_ system, interstitial sites are located in the AO layer, in which A_2_BO_4_ oxides can accommodate an excess oxygen as an interstitial oxygen defect. Oxygen vacancies can also be formed by appropriate doping in the system. Consequently, the oxygen transport properties of A_2_BO_4_ oxides can be strongly influenced by either oxygen interstitials or oxygen vacancies. In addition, different types of oxygen defects affect the oxidation states of transition metal cations of A_2_BO_4_ oxides as a result of changes in oxygen stoichiometry, and they thus lead to different lattice expansions.

A fundamental understanding of oxygen migration in A_2_BO_4_ oxides is therefore necessary to optimize superior oxygen ion transport for the development of applications in electrochemical energy devices, chemical sensors, and oxygen permeation membranes. This review aims to briefly review oxygen migration mechanisms depending on oxygen stoichiometry in the first member of the RP series (*n* = 1) and provide a rational idea of how to control oxygen ion migration in A_2_BO_4_ oxides. We first discuss the mechanisms of oxygen ion migration in oxygen-excess and oxygen-deficient A_2_BO_4_ oxides, providing an overview of reported experimental and theoretical results. We then cover in detail the effects of three factors—cation substitution, crystallographic orientation, and strain—on oxygen migration in A_2_BO_4_ oxides. The thermochemical stability of A_2_BO_4_ oxides is also briefly reviewed in a separate section. Finally, we provide a perspective on open questions on controlling oxygen ion migration in A_2_BO_4_ oxides and suggest some potential research directions to facilitate oxygen ion migration.

## 2. Oxygen Migration Mechanisms in Ruddlesden–Popper Oxides

In contrast to ABO_3_ oxides, which are generally known as oxygen-deficient perovskites because oxygen vacancies are their dominant anion defect, RP oxides can be both oxygen-deficient and oxygen-excess, depending upon their majority oxygen defects. In the case of oxygen-deficient RP oxides, oxygen nonstoichiometry (δ) arises from oxygen vacancies, whereas oxygen interstitials result in oxygen hyperstoichiometric RP oxides. Therefore, oxygen ion migration in RP oxides can occur via mechanisms associated with either oxygen vacancies or oxygen interstitials. The defect processes in RP oxides are dominated by anion Frenkel disorder [51,55,56] and can be expressed as

*O_O_^x^* ⇔ *V*_O_¨ + *O*_i_*”*. (1)

The oxygen diffusion coefficient *D*_O_ can be expressed in terms of the defect concentrations and their diffusivities as [57]

*D*_O_ = *D*_V_[*V*_O_¨] + *D*_i_[*O*_i_*”*] (2)

where *D*_V_ and *D*_i_ are the vacancy and interstitial diffusion coefficients, respectively. [*V*_O_¨] and [*O*_i_*”*] represent the concentrations of oxygen vacancies and oxygen interstitials, respectively.

### 2.1. Oxygen-Excess RP Oxides

Many studies have shown oxygen ion transport properties in oxygen-excess RP oxides, mostly focusing on La_2_NiO_4+δ_ (LNO_214_), which can accommodate excess oxygen by the incorporation of oxygen interstitials in the lattice [50,58,59,60,61]. Owing to the structural feature of RP oxides made of a stacking sequence of A_2_O_2_ and BO_2_ layers, LNO_214_ is a polar material and has an electric field gradient between the La_2_O_2_^2+^ and NiO_2_^2−^ layers. Accordingly, the Coulomb potential leads to an expected large anisotropy in the oxygen transport by preventing the interstitial O^2−^ ions from leaving the La_2_O_2_^2+^ layer [50,62]. It is well known that oxygen diffusion along the *a-b* planes is much faster than in the *c*-direction [49,50,63,64,65]. Using atomistic simulation calculations, Minervini et al. [55] determined that the intrinsic disorder for LNO_214_ is anion Frenkel disorder with the oxygen vacancy residing on the equatorial site. According to the authors, oxygen ion migration in LNO_214_ occurs via an interstitialcy (or push–pull) mechanism, whereby at an apical site, the oxygen vacancy formed by the movement of apical oxygen into the interstitial sites in the rock salt layer is filled by a nearby interstitial oxygen. Consequently, the creation of Frenkel anionic defects, which are oxygen interstitials and oxygen vacancies, leads to additional oxygen content. Later calculations by Cleave et al. [66] showed that the most energetically favorable mechanism for oxygen migration in LNO_214_ is the vacancy mechanism in the *a-b* plane, where the equatorial oxygen vacancies are prone to migrate in the equatorial plane. Frayret et al. [67] also demonstrated an interstitial diffusion mechanism in LNO_214_ using density functional theory (DFT). The authors proposed that charge transfer phenomena play a minor role in the oxygen migration process, reporting that equatorial oxygen vacancies are energetically more favorable than apical oxygen vacancies to form Frenkel-type defects. More recently, molecular dynamics (MD) studies [63] showed that an interstitialcy mechanism—involving an interstitial oxygen, a neighboring apical oxygen, and an appropriate equatorial oxygen site—plays an important role in oxygen transport in LNO_214_.

**Figure 2:**
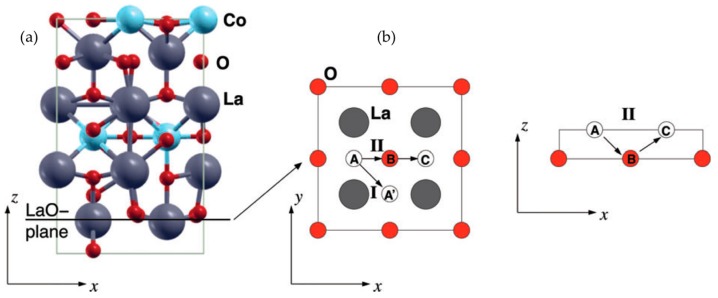
(**a**) Relaxed configuration of the La_2_CoO_4+δ_ model. Circled region represents an interstitial oxygen atom; and (**b**) migration paths in simulation. (**I**) indicates an interstitial migration mechanism where an oxygen interstitial atom at the A site directly hops to the adjacent interstitial site A’; (**II**) shows an interstitialcy migration mechanism. An oxygen interstitial atom at the A site kicks the oxygen atom at the B site out of the LaO plane, sending it to the next interstitial site C, and placing itself on the B site on the LaO plane. Reprinted from Ref. [70] with permission of The Royal Society of Chemistry

Similarly, MD simulations by Parfitt et al. [68] of Pr_2_NiO_4+δ_ (PNO_214_) showed that oxygen ion migration is also highly anisotropic, occurring via an interstitialcy mechanism. The authors reported that the degree of excess oxygen strongly influences the activation energy for oxygen migration in PNO_214_. Similar to the case for LNO_214_, calculations for oxygen migration in La_2_CuO_4+δ_ (LCuO_214_) by Allan et al. [69] showed that oxygen vacancies at the equatorial site are more favorable for migration in the equatorial plane, compared with interstitial oxygen. According to the calculated results from Kushima et al. [70], oxygen migrates dominantly via an interstitialcy mechanism in La_2_CoO_4+δ_ (LCO_214_) (Figure 2). Kushima et al. performed both DFT and MD simulations, demonstrating that although the predicted activation energies for oxygen migration in DFT and MD are different from each other, oxygen migration via an interstitialcy migration mechanism has a significantly lower activation energy than migration via direct interstitial site exchange. The oxygen migration energies for oxygen-excess RP oxides are summarized in Table 1.

### 2.2. Oxygen-Deficient RP Oxides

The addition of acceptors to oxygen-excess RP oxides can drive the stoichiometry of RP oxides into oxygen deficiency [46,71,72]. For example, the substitution of an aliovalent cation such as Sr^2+^ (1.31 Å) for a cation such as La^3+^ (1.22 Å) on the A site leads to an increase of oxygen deficiency in RP oxides. Table 2 shows a summary of oxygen stoichiometry as a function of the strontium (Sr) content in RP oxides. Opila et al. [56] proposed the defect model for Sr-doped cuprates (La_2−x_Sr_x_CuO_4−δ_), showing anisotropic oxygen transport properties. Owing to the formation of Sr_La_’ defects, charge compensation can occur by either electron holes or oxygen vacancies.

2Sr_La_’ + *V*_O_¨ = {(Sr_La_’)_2_*V*_O_¨}^x^. (3).

Then, the overall electroneutrality can be given by

*p* + 2[*V*_O_¨] = *n* + 2[*O*_i_*”*] + [Sr_La_’]. (4)

Opila et al. [56] suggested that a vacancy mechanism is responsible for oxygen migration in La_2−x_Sr_x_CuO_4−δ_ (LSCuO_214_) at low Sr contents (x < 0.07). MD simulations by Mazo et al. [73] of LSCuO_214_ (Sr = 0.37) demonstrated that a strong anisotropy of the crystal structure can lead to different oxygen transport properties in (La,Sr)O blocks and CuO_2_ layers, the latter being more favorable for oxygen transport via a conventional vacancy migration mechanism. More recently, Savvin et al. [74] also investigated the oxygen diffusion processes in LSCuO_214_ (Sr = 0.15 and 1.0) via MD simulations, demonstrating that oxygen migration occurs mainly in CuO_2_ layers as a result of a hopping mechanism. The authors proposed that, by jumping to the nearest position or along the CuO_2_ layers, oxygen can migrate in LSCuO_214_. In addition, they also pointed out that although the migration energy of oxygen vacancies at apical sites is much lower than that at the equatorial sites, oxygen migration along the *c*-direction can occur only with great difficulty, as vacancies at the apical sites must pass through the equatorial plane, resulting in an increase in the activation energy.

**Figure 3:**
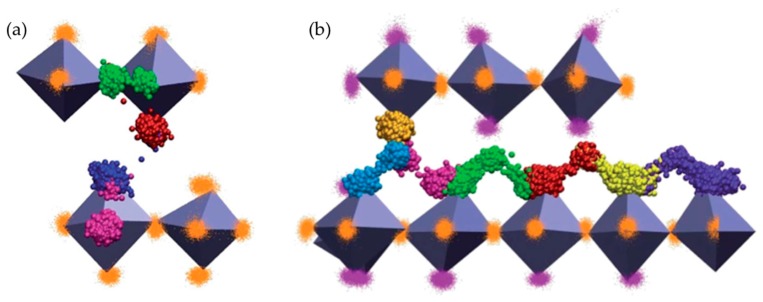
(**a**) 3D representation of oxygen vacancy migration between equatorial and apical positions within a CoO_6_ octahedron (pink and red), between equatorial positions (green) and between apical positions belonging to separate layers (blue) in La_10.8_Sr_1.2_CoO_3.9_; (**b**) 3D representation of oxygen interstitial migration in La_1.2_Sr_0.8_CoO_4.1_. Each oxygen involved in the migration event is represented by a different color. Spheres of the same color indicate the positions occupied by a specific atom over the simulation time. Reprinted from Ref. [79] with permission of The Royal Society of Chemistry.

The oxide ion migration mechanisms of La_2−x_Sr_x_CoO_4±δ_ (LSCO_214_) for high Sr levels (x = 0.8 and 1.2) were reported by Tealdi et al. [79] via MD simulations (Figure 3). They proposed that oxygen diffusion in oxygen-deficient La_0.8_Sr_1.2_CoO_3.9_ is mainly attributable to the migration of oxygen vacancies within the perovskite layer, whereas an interstitialcy mechanism is dominant for interstitial oxygen transport in oxygen-excess La_1.2_Sr_0.8_CoO_4.1_. They further suggested that it is also possible for oxygen vacancies to migrate through long-range paths between adjacent layers. Schroeder et al. [45] investigated the oxygen transport property of polycrystalline La_2−x_Sr_x_NiO_4+δ_ (LSNO_214_, x = 0.5) samples by experimental results for the oxygen permeation flux. The authors claimed that oxygen migration in LSNO_214_ with Sr = 0.5 is mediated mainly by interstitial oxygens, occurring parallel to the layers of the structure, although the contribution of vacancy migration to the oxygen permeation process cannot be ruled out.

## 3. Control of Oxygen Migration in RP Oxides

As discussed earlier, oxygen migration in RP oxides is strongly dependent on the defect concentrations, which can change the degree of oxygen stoichiometry. In general, substitutions on the A or B sites of RP oxides are known to affect the oxygen stoichiometry, leading to a change in the oxygen transport properties [64]. In addition, the anisotropic nature of oxygen migration in RP oxides can cause two different behaviors in oxygen transport [50]. Furthermore, recent computational and experimental studies have shown that epitaxial strain in a thin film, induced by lattice mismatch, can alter oxygen migration in binary oxides [80,81,82,83,84] and ABO_3_ perovskite oxides [7,85,86]. In the following subsections, we focus on how these three factors, i.e., cation substitution, crystallographic orientation, and strain, can influence oxygen migration in RP oxides.

### 3.1. Influence of Cation Substitutions on Oxygen Migration

Cation substitutions have been widely used to facilitate the oxygen ion diffusion and oxygen surface exchange kinetics in ABO_3_ perovskites such as lanthanum (La)-based oxides, in which a hopping mechanism along the BO_6_ octahedron edge governs the oxygen vacancy migration [87,88]. It is well known that substituting Sr for La on the A-site of LSC_113_ results in an increase in oxygen vacancies and thus enhances the oxide ion diffusivity [10,89,90,91,92,93]. In addition, doping with different-size cations in ABO_3_ perovskites can also facilitate oxygen migration owing to a reduction in the ion migration energy [84]. The effect of Sr substitution on the activation energy for oxygen diffusion in LSC_113_, reviewed by Berenov et al. [5], is that the activation energy for oxygen diffusion decreases with increasing Sr content owing to the reduced vacancy formation energy. Cater et al. [15] compared the oxygen diffusivity between Sr-doped cobaltites and Sr-doped manganites, concluding that B-site cations with a lower valence state increase in oxygen diffusivity. In the case of RP oxides, however, the effect of cation substitutions on oxygen migration is poorly understood, compared with the case of ABO_3_ perovskites. In this section, we review oxygen diffusion depending upon either A-site or B-site substitution in the most widely studied RP nickelates, cuprates, and cobaltites.

#### 3.1.1. Effect of Substitutions on the A-Site

**Figure 4:**
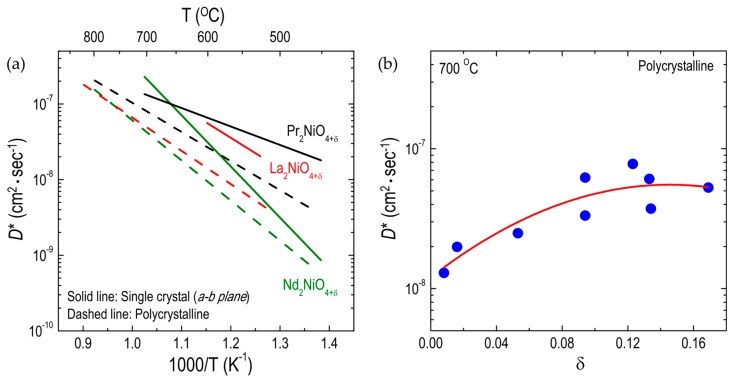
Arrhenius plots of oxygen tracer diffusivity (D*) for different RP oxides: (**a**) Effect of A-site substitutions on D* for A_2_NiO_4+δ_ (A = Pr, La, and Nd) single crystalline and polycrystalline oxides: single crystal (Ref. [49,97]) and polycrystalline (Ref. [51]); (**b**) Dependences of D* on the oxygen nonstoichiometry in A_2_NiO_4+δ_ (A = Pr, La, and Nd) polycrystalline oxides (Ref. [51]).

LNO_214_ is one of the most intensively studied RP oxides in terms of oxygen transport properties [51,61,72,94,95]. Using the isotope exchange depth profile method, the oxygen self-diffusion coefficient (D*) values of polycrystalline LNO_214_ samples were found to be higher than those of LSCF_113_ and similar to the diffusivity in LSC_113_ [53,96]. Later, Boehm et al. [51] reported a similar oxygen diffusivity for LNO_214_, showing the oxygen diffusivity of PNO_214_ and Nd_2_NiO_4+δ_ (NNO_214_) polycrystalline samples as well (Figure 4a). In this study, the diffusivity of PNO_214_ was found to be the highest, with the lowest activation energy among the three RP nickelates in the temperature range of 550 to 850 °C. Recently, the D* values for the same RP nickelates were examined using single crystals [49,50,97] and showed a similar trend to that reported for polycrystalline samples (Figure 4a). The D* values along the *a-b* plane for PNO_214_ single crystals were found to be higher, with a significantly lower activation energy (0.67 eV), compared with NNO_214_ single crystals at low temperatures [49]. In the case of LNO_214_ single crystals, the D* values along the *a-b* plane and the activation energy (0.88 eV) for the diffusion were found to be comparable to the data observed in polycrystalline LNO_214_ [50]. The diffusivity measurements in the *a-b* plane for LNO_214_ single crystals were repeated by Burriel et al. [97]; their findings agreed well with the previous data [50]. They also demonstrated that the D* values along the *a-b* plane for LNO_214_ single crystals are comparable to those for PNO_214_ single crystals. Considering the oxygen nonstoichiometry (δ) determined in air at room temperature in PNO_214_ (δ = 0.21 [51]), LNO_214_ (δ = 0.13 [51]), and NNO_214_ (δ = 0.22 [51]), it was proposed that the larger δ value resulted in higher oxygen migration among the three RP nickelates, which have a sharp maximum D* value at a certain δ value (≈0.125) as a result of increased defect interactions [49,51] as shown in Figure 4b. However, this cannot explain the difference in oxygen transport in PNO_214_ compared with NNO_214_ despite the similar δ. Recently, Bassat et al. [49] suggested that an interplay between lattice dynamics and structural instabilities in RP structures may also lead to a difference in the oxygen migration between PNO_214_, LNO_214_, and NNO_214_, which may be responsible for the difference in oxygen transport between PNO_214_ and NNO_214_. Further investigation is needed to clarify the relationship between δ and D*. Activation energies for the oxygen diffusivities of various RP oxides are summarized in Table 3.

**Figure 5:**
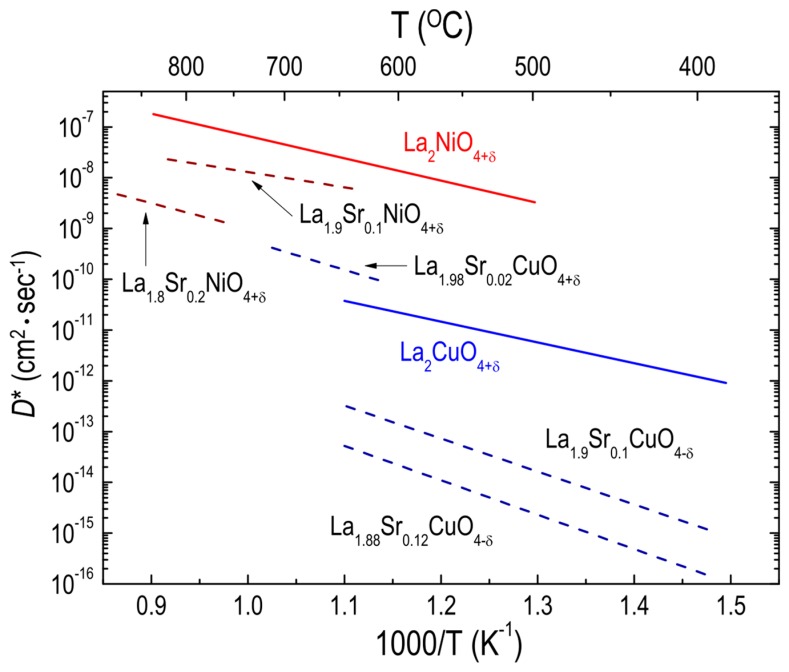
Arrhenius plots of oxygen tracer diffusivity (D*) for different RP oxides. Effect of Sr substitution on the A site on D* for La_2−x_Sr_x_CuO_4+δ_ (Ref. [56]) single crystal and La_2−x_Sr_x_NiO_4+δ_ (Ref. [51]) polycrystalline oxides.

Investigations of the effect of A-site substitution on the oxygen transport properties of RP oxides have focused mainly on two material systems, LSNO_214_ and LSCuO_214_ [42,53,96,99,100]. Routbort et al. [100] measured the D* values for polycrystalline LSCuO_214_ oxides with a Sr content from 0.1 to 0.2, concluding that the decreased oxygen diffusion of LSCuO_214_ with increasing Sr content may be attributed to the immobilization of oxygen vacancies resulting from the addition of Sr. Using single crystals of LSCuO_214_ with Sr content from 0 to 0.12, Opila et al. [56] demonstrated the same trend of decreased oxygen diffusivity as Sr content increased. Similarly, LSNO_214_ polycrystalline oxides [51] were found to show a decrease in oxygen diffusivity with increasing Sr content. Likewise, Sr substitutions on the A-site of LSNO_214_ resulted in a reduction in the oxygen permeability [72]. The data are shown as part of the compilation of data in Figure 5. As discussed earlier, the ionic radii of Sr^2+^ and La^3+^ (1.31 Å and 1.22 Å, respectively [101]) are quite different. Therefore, substituting a larger cation for a cation ion on the A site in RP oxides leads to a structural stress relaxation, which in turn decreases the number of additional oxygen atoms required to stabilize the structure [102]. Consequently, the oxygen diffusion can decrease, owing to the decreased amount of additional oxygen. On the contrary, decreased δ may not always lead to a reduction in oxygen migration in RP oxides if the A-site cation is substituted by a cation with the same ionic radius and a smaller oxidation state, in accordance with a previous study on oxygen diffusion in calcium (Ca)-doped NNO_214_ (Ca = 0.2) [51]. One point to note from Figure 5 is that the oxygen diffusivity for LSCuO_214_ increases with increasing Sr content (Sr = 0.02) relative to LCuO_214_, whereas a further increase in the Sr content (Sr > 0.02) results in decreased oxygen diffusivity. Opila et al. [56] proposed that initially increased oxygen vacancies due to the Sr additions (Sr = 0.02) result in enhanced oxygen diffusivity, while further Sr additions (Sr > 0.02) cause oxygen vacancy ordering, decreasing the oxygen diffusivity. Although the trend toward reduction of the oxygen diffusivity with increasing Sr content in RP cuprates and nickelates has also been shown by other groups [61,95,99], most studies of the effects of Sr substitution on oxygen diffusivity in RP oxides have used a very narrow range of Sr content, from 0.0 to 0.2.

#### 3.1.2. Effect of Substitutions on the B-Site

**Figure 6:**
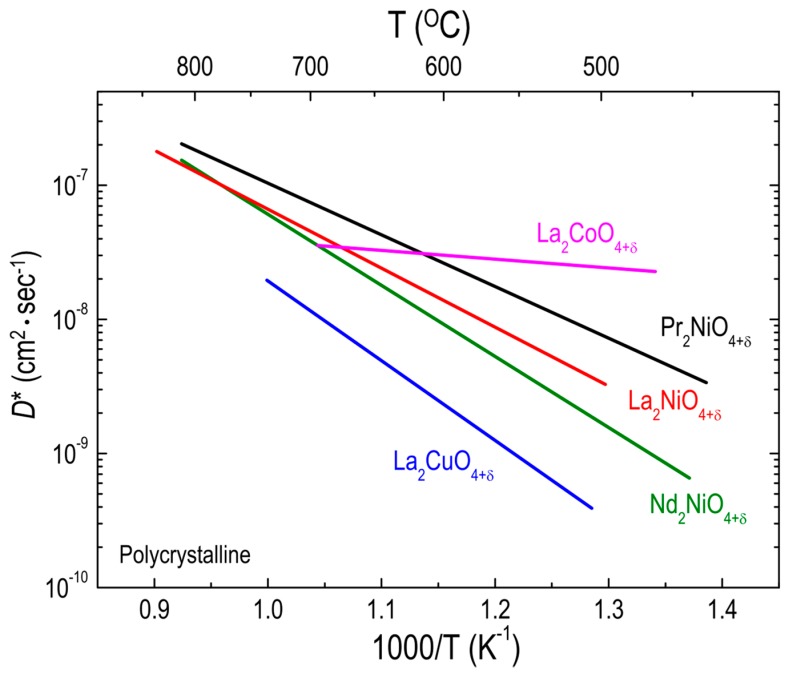
Arrhenius plots of oxygen tracer diffusivity (D*) for different RP oxides. Effect of B-site substitutions on D* for La_2_BO_4+δ_ (B = Co, Ni, and Cu) polycrystalline oxides: La_2_CoO_4+δ_ (Ref. [77]), La_2_NiO_4+δ_ (Ref. [51]), and La_2_CuO_4+δ_ (Ref. [98]). The D* values for Pr_2_NiO_4+δ_ and Nd_2_NiO_4+δ_ (Ref. [51]) are also plotted for comparison.

Munnings et al. [77] achieved a remarkably low activation energy (0.13 eV) for oxygen diffusion of polycrystalline LCO_214_ samples below 700 °C, above which LCO_214_ can be easily decomposed into LaCoO_3_ and La_2_O_3_. According to the comparison of the D* values for La_2_BO_4+δ_ (B = Co [77], Ni [51], and Cu [98]) polycrystalline samples in Figure 6, LCO_214_ is the best material, with the highest oxygen diffusivity and the lowest activation energy in the temperature range of 450–700 °C where the oxygen diffusivity of LNO_214_ is higher than that of LCuO_214_. Moreover, the D* values for LCO_214_ appear much higher than those for both PNO_214_ and NNO_214_ below 630 °C. This fact implies that substitution on the B site has more influence on the oxygen ion transport in RP oxides than does A-site substitution. Considering that the concentration of oxygen interstitials in LCuO_214_ is much lower than in either LCO_214_ or LNO_214_, owing to the direct correlation between δ and B^3+^ content [103,104,105], a lower oxygen diffusivity for LCuO_214_ can be expected, as discussed in Section 3.1.1. However, the δ value of LCO_214_ (δ = 0.13 [77]) is highly comparable to that of LNO_214_ (δ = 0.13 [51]) and much smaller than that of PNO_214_ (δ = 0.21 [51]) and NNO_214_ (δ = 0.22 [51]) although the D* value for LCO_214_ is the highest among these materials at low temperatures. Although the defect concentration above a certain value may decrease the oxygen transport as a result of increased defect interactions, as discussed earlier, this possibility cannot address the LCO_214_ case. Thus, other factors, such as oxygen defect formation energy, may contribute to the oxygen diffusivity of RP oxides. Recently, Lee et al. [106] proposed that the oxygen 2*p* band center relative to the Fermi energy is an effective descriptor to predict the oxygen surface exchange as well as the activation energy for oxygen transport in RP oxides. According to this work, the calculated interstitial formation energies of La_2_BO_4+δ_ (B = Co, Ni, and Cu) strongly depend on the B cation in the sequence LCuO_214_ > LNO_214_ > LCO_214_, which corresponds to the trend of B-cation–dependent oxygen diffusivity. More recently, Xie et al. [107] also calculated the formation energy of oxygen interstitials in the same RP oxide systems, demonstrating that, regardless of δ, the formation energy of oxygen interstitials decreases with decreasing the atomic number of the B cation, which is in good agreement with the data reported by Lee et al. [106].

**Figure 7:**
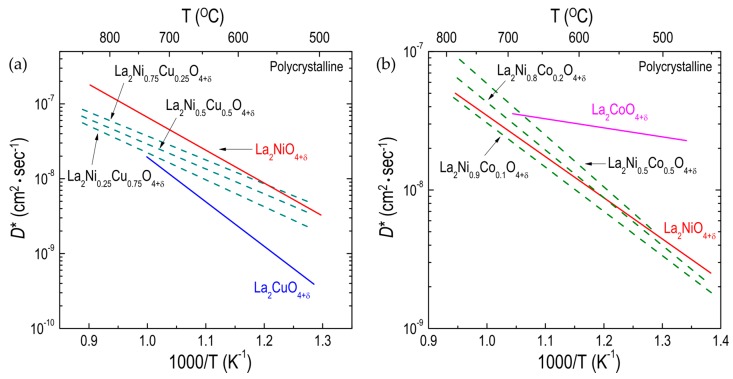
Arrhenius plots of oxygen tracer diffusivity (D*) for different RP oxides: (**a**) effect of Ni substitution on the B site on D* for La_2_Ni_1−x_Cu_x_O_4+δ_ (Refs. [51,98]) polycrystalline; and (**b**) effect of Ni substitution on the B site on D* for polycrystalline La_2_Ni_1−x_Co_x_O_4+δ_ (Refs. [77,94]) polycrystalline oxides.

Boehm et al. [51,98] measured the oxygen diffusivity for polycrystalline La_2_Ni_1−x_Cu_x_O_4+δ_ (x = 0, 0.25, 0.5, 0.75, and 1.0), showing that the oxygen diffusion for La_2_Ni_1−x_Cu_x_O_4+δ_ decreases slightly as the copper (Cu) content increases (Figure 7a). The effect on oxygen diffusion of substituting cobalt (Co) for nickel (Ni) in La_2_Ni_1−x_Co_x_O_4+δ_ has been evaluated by several different groups [51,77,94,108]. Kilner et al. [94] found that the activation enthalpy for oxygen migration in polycrystalline La_2_Ni_1−x_Co_x_O_4+δ_ slightly increased with increasing Co content. Although the D* values for LCO_214_ have been measured by different groups, the overall trend is that the degree of change in oxygen diffusivity from partially substituting Co for Ni in La_2_Ni_1−x_Co_x_O_4+δ_ is not significant, as shown in Figure 7b. The number of interstitial oxides of La_2_Ni_1−x_Cu_x_O_4+δ_ was found to slightly decrease with increasing Cu content [98], and partially substituting Co for Ni changed a small amount of δ in La_2_Ni_1−x_Co_x_O_4+δ_ [94,108]. Therefore, this substitution may result in small changes in the D* values for La_2_Ni_1−x_B’_x_O_4+δ_ when Ni is partially substituted by either Cu or Co. However, compared with the strong dependency of the D* of La_2_BO_4+δ_ (B = Co, Ni, Cu) on the B-site cation, as discussed earlier, the degree of change in the oxygen diffusivity resulting from partial substitution on the B site is almost negligible. That finding suggests that the partial substitution of the B-site cation by other cations has a negligible effect on oxygen ion transport in La_2_B_1−x_B’O_4+δ_. It was assumed that partial substitution on the B site does not lead to significant changes in the oxygen-cation bonds in the BO_6_ octahedra and thus may have only a negligible effect on the oxygen diffusivity of La_2_Ni_1−x_B_x_O_4+δ_ [51].

### 3.2. Influence of Crystallographic Orientation on Oxygen Migration

As discussed earlier, the anisotropy of oxygen migration is one of the intrinsic properties of RP oxides owing to its structural feature. In the case of polycrystalline materials, the anisotropy is averaged; therefore, the D* value depends on the connectivity between grains. Introducing single crystals or epitaxial thin films has enabled investigation of the anisotropic nature of oxygen transport properties in RP oxides. For example, Burriel et al. [58] reported that the oxygen diffusion and oxygen surface exchange kinetics in the *a-b* plane are faster than those along the *c*-direction in epitaxial LNO_214_ thin films. Lee et al. [102] demonstrated that substituting Sr for La in LSNO_214_ thin films can result in the structural reorientation of the films because of the reduction in the surface energy of the (001) surface, reporting the anisotropic oxygen surface exchange kinetics. However, Chen et al. [109] did not obtain any orientation-dependent surface exchange kinetics in epitaxial La_2−x_Sr_x_CoO_4+δ_ (LSCO_214_, Sr = 0.25) thin films, in contrast to the observation that oxygen diffusion is orientation dependent.

**Figure 8:**
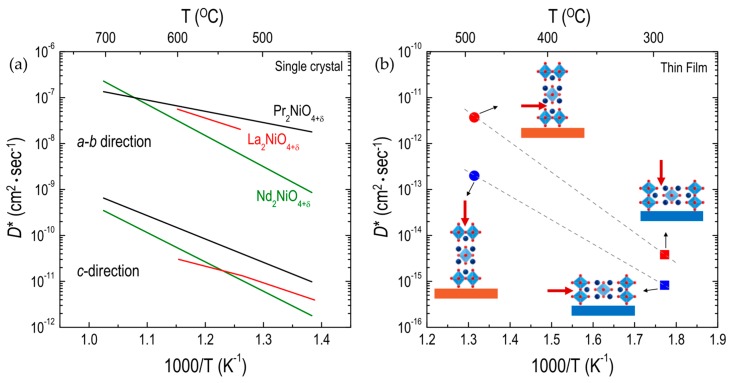
Arrhenius plots of oxygen tracer diffusivity (D*) for different RP oxides: (**a**) D* along the *a-b* plane and *c*-direction in A_2_NiO_4+δ_ (A = Pr, La, and Nd, Refs. [49,97]) single crystalline oxides; and (**b**) D* along the *a-b* plane and *c*-direction in the (001)-oriented (circle) and the (100)-oriented (square) La_1.75_Sr_0.25_CoO_4+δ_ thin films [109].

Anisotropic oxygen migration in RP oxides has been investigated, mainly focusing on RP nickelate single crystals, by Bassat and co-workers [49,50,97]. As shown in Figure 8a, three different RP nickelates (i.e., PNO_214_, LNO_214_, and NNO_214_) show a large anisotropy in the oxygen diffusion. In the temperature range of 450–700 °C, the oxygen diffusion along the *a-b* plane is about three orders of magnitude higher than that along the *c*-direction for all materials. Bassat et al. [49] suggested that the *a-b* plane diffusion process is dominant in polycrystalline samples, as the D* values along the *a-b* plane for single crystals are comparable to those for polycrystalline samples (Figure 4a). In addition, they found that the D* values along the *a-b* plane in RP nickelates are generally larger than those in ABO_3_ perovskites. Claus et al. also showed [99] anisotropic oxygen diffusion in single-crystal LCuO_214_, where the D* values along the *a-b* plane are two orders of magnitude higher than those along the *c*-direction.

The effect of crystallographic orientation on the oxygen ion transport can be more clearly seen in thin films. Recently, Chen et al. [109] successfully fabricated LSC_214_ (Sr = 0.25) thin films grown in two different crystallographic orientations, i.e., (100) and (001), using (100) LaSrAlO_4_ (LSAO) and (001) SrTiO_3_ (STO) substrates. They demonstrated that the oxygen diffusivity along the *a-b* plane in the (100)-oriented LSC_214_ film was three orders of magnitude lower than that in the (001)-oriented LSC_214_ film (Figure 8b). Moreover, the D* value along the *c*-direction in the (001)-oriented LSC_214_ film was found to be one order of magnitude higher than that along the *a-b* plane in the (100)-oriented LSC_214_ film.

### 3.3. Influence of Strain on Oxygen Migration

The use of epitaxial strain induced in a thin film by lattice mismatch with a substrate has been intensively studied for control of oxygen migration and the formation of oxygen defects in binary oxides [80,81,82,83,110,111,112,113,114] and ABO_3_ perovskite oxides [7,85,86,115,116,117,118,119]. Theoretical studies [110,112] have shown that tensile (compressive) strain can decrease (increase) the formation energy of oxygen vacancies in CeO_2_. De Souza and co-workers [80] calculated the activation energies for oxygen migration in strained CeO_2_, demonstrating that the oxygen migration in tensile strain is remarkably faster than in compressive strain. Similar to research on binary oxides, Mayeshiba and Morgan [85] predicted the oxygen migration barriers in LaMO_3_ (M = Sc, Ti, V, Cr, Mn, Fe, Co, Ni, and Ga) perovskite oxides under tensile strain via DFT calculations. They demonstrated that tensile strain can significantly reduce the oxygen migration barriers, resulting in enhanced oxygen diffusion (one order of magnitude) at 500 °C compared with the diffusion under unstrained conditions. Kubicek et al. [7] synthesized LSC_113_ thin films on STO and LaAlO_3_ (LAO) substrates, which introduce tensile and compressive strain states, respectively, into the LSC_113_ films. They demonstrated that the D* values for tensile-strained LSC_113_ are two orders of magnitude higher than those for compressive-strained LSC_113_, postulating that an increase in the oxygen vacancies due to tensile strain may contribute to the enhanced oxygen diffusion. Similarly, SrCoO_3−δ_ (SCO_113_) thin films revealed a sensitive response to strain, modifying the oxygen diffusion and activation [86,118].

**Figure 9:**
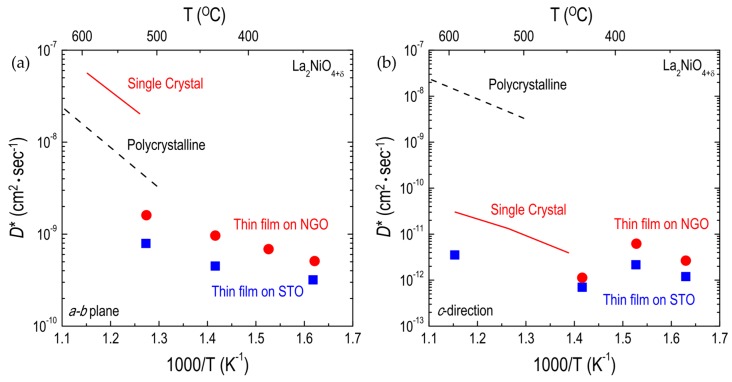
Arrhenius plots of oxygen tracer diffusivity (D*) for La_2_NiO_4+δ_ films: (**a**) D* along the *a-b* plane; and (**b**) along the *c*-direction for La_2_NiO_4+δ_ films on SrTiO_3_ (STO) and NdGaO_3_ (NGO). Thin film [58], single-crystal [97], and polycrystalline [51] data are comparatively shown.

In contrast to binary oxides and ABO_3_ perovskite oxides, very few studies have reported the effects of strain on oxygen diffusion and surface exchange in RP oxides. Using pulsed laser deposition, Burriel et al. [58] fabricated (001)-oriented LNO_214_ films on STO and NdGaO_3_ (NGO) substrates, which led to bi-axial tensile and compressive strain states, respectively, along the *a-b* plane. They evaluated the oxygen diffusion and surface exchange kinetics for tensile- and compressive-strained LNO_214_ films, concluding that both compressive and tensile strain led to a reduction in the oxygen diffusivity in the first 175–200 nm of the film, whereas neither strain affected the oxygen surface exchange. Therefore, the D* values for the LNO_214_ films were found to be lower than those for either polycrystalline or single-crystal LNO samples regardless of the diffusion direction, as shown in Figure 9. However, Lee et al. [52] later showed that in (100)-oriented LNO_214_ thin films, tensile strain along the *c*-direction can lead to an increase in the driving force to form interstitial oxygen atoms in LNO_214_, resulting in enhanced oxygen surface exchange kinetics. Based on this work, the facilitated oxygen migration in tensile-strained LNO_214_ thin films can be inferred to show that more oxygen interstitials in RP nickelates can lead to enhancement of the oxygen diffusivity, as discussed in Section 3.1.1. More recently, Tsvetkov et al. [120] compared the δ values of tensile- and compressive-strained NNO_214_ thin films, demonstrating that the larger δ in tensile-strained NNO_214_ can enhance the oxygen surface exchange kinetics relative to those of compressive-strained NNO_214_ thin films. From this work, it can also be postulated that tensile-strained NNO_214_ would show increased oxygen migration. The discrepancy in the effects of strain on oxygen surface exchange kinetics in RP films, mentioned earlier, may be attributed to the fact that the critical thickness required to maintain an acceptable strain state is fairly smaller than what Burriel et al. [58] used. However, the effect of strain on oxygen migration in RP oxides needs further investigation.

## 4. Control of Oxygen Stability in RP Oxides

To implement oxide materials for practical applications, such as SOFCs and oxygen permeation membranes operated under a wide range of temperatures and oxygen partial pressures, oxide materials should possess high thermal and chemical stability. In the case of ABO_3_ perovskite oxides, a vast number of studies [1,20,21,26,28,29,30,32,33,121,122,123,124,125,126,127,128,129] related to the chemical stability and thermal expansivity of ferrites and cobaltites have been reported, as both material systems have high electronic and ionic conductivities useful in many applications [1,130,131,132]. However, ferrites and cobaltites exhibit high thermal expansion coefficients, as well as low chemical stability, because both materials can easily lose lattice oxygen, resulting in unfavorable expansion of the lattice in reducing atmospheres [28,133]. Here, we briefly review RP oxides based on nickelates, cuprates, and cobaltites with respect to their thermal and chemical stability.

LNO_214_ was found to have a structural transition from the *Fmmm* orthorhombic to the *I4/mmm* tetragonal structure between room temperature (RT) and 150 °C [134]. Huang et al. [135] also observed a structural transition (i.e., orthorhombic-tetragonal transition) of LNO_214_ between RT and 150 °C, above which no phase transition occurred. LSNO_214_ is expected to have a different thermal expansion behavior owing to a change in δ from adding Sr content [42,136,137,138]. Although the tetragonal structure of LSNO_214_ (Sr = 0.2 and 0.4) was retained over the temperature range between 600 and 900 °C, thermal expansion coefficients increased with increasing Sr content [138]. Makhnach et al. [136] reported significantly different high-temperature behavior of LSNO_214_ (Sr = 1.4) due to evolution of the Ni valence from Ni^2+^ to Ni^3+^ compared with LNO_214_. They found significantly larger variations of cell parameters *a* and *c* above 600 °C. Thermal expansion coefficients of ABO_3_ and A_2_BO_4_ oxides are shown in Table 4. The effect of partial substitution on the B-site in RP nickelates was investigated by Kharton and co-workers [139,140]. In these studies, the equilibrium chemical strains induced by the oxygen hyperstoichiometry variations in La_2_Ni_1−x_B_x_O_4+δ_ (B = Fe, Co, and Cu) were found to be very low relative to ABO_3_ ferrites and cobaltites owing to a strongly anisotropic expansion of the RP structures [139]. However, at low oxygen partial pressure, the decomposition of Ni-containing phases was observed, which indicates that the partial substitution of other transition metal cations for Ni had no effect on the stability of LNO_214_ [140].

Kanai et al. [75] reported that the oxygen partial pressure for the decomposition of LSCuO_214_ is strongly dependent on the Sr contents. According to this work, although the decomposition of LSCuO_214_ is not dependent on oxygen partial pressure below Sr = 0.05, the dependence of decomposition on the oxygen partial pressure increases above Sr > 0.1. This suggests that an abrupt variation in the thermodynamic behavior of LSCuO_214_ occurs in the region between Sr = 0.05 and Sr = 0.1. In terms of thermal stability, LSCuO_214_ with Sr = 0.6 was found be more stable than LSCuO_214_ with Sr = 0.2 during heating in a vacuum because the higher Sr content prevented Cu^+^ formation and oxygen removal [78].

LSCO_214_ (0.5 ≤ Sr ≤ 1.5) showed stable reduction behaviors during heating and cooling between 300 and 1500 °C [46], whereas substituting a B cation for Co resulted in a slight increase in thermal expansion coefficients [145]. However, LCO_214_ without the Sr content was found to decompose into LaCoO_3_ and La_2_O_3_ above 700 °C [77], as discussed in Section 3.1.2. In addition, RP cobaltites and nickelates were found to have greater thermal stability than ABO_3_-based cobaltites and ferrites [27].

## 5. Conclusions

Developing high-performance energy and environmental devices, such as SOFCs, oxygen permeation membranes, and chemical sensors, requires discovering new oxide materials with high oxygen mobility and stability. In this regard, RP oxide materials (A_n+1_B_n_O_3n+1_ with *n* = 1) have been considered as alternative materials to ABO_3_ perovskite oxides. In this review, we have briefly overviewed the fundamental mechanisms of oxygen ion migration, approaches to control oxygen transport, and stability in RP oxides, focusing on the A_2_BO_4_ system. A clear distinction between RP oxides and ABO_3_ oxides is that RP oxides can be both oxygen-deficient and oxygen-excess depending on the δ, which can determine whether the oxygen migration mechanism is driven by interstitialcy or vacancy. In addition, RP oxides have an anisotropic structure, which leads to highly anisotropic diffusion properties. Understanding how multiple factors associated with defect formation and structural features affect oxygen transport can provide insights into developing novel approaches to control oxygen migration in RP oxides.

The three factors discussed in this review—cation substitution, crystallography orientation, and strain—have an apparent influence on oxygen ion migration, remaining open questions with regard to the control of oxygen migration in RP oxides. To address these questions, two approaches are promising for further investigation. First, the development of descriptors, such as the metal *d*-band center and oxygen *p*-band center, by DFT calculations and MD simulations helps predict the trends associated with the changing nature of the A- and B-site cations. We discussed how the oxygen *p*-band center of lanthanum-based RP oxides correlates with the formation energy of oxygen defects. This concept can be extended to a series of various RP oxides, leading to an acceleration in the discovery of the best materials for applications requiring high oxygen mobility. High-throughput computational and combinatorial screening will also be helpful in identifying optimized materials. The second is the use of epitaxial thin films, which can be fabricated by pulsed laser epitaxy, sputtering, or molecular beam epitaxy. RP series oxides show strong anisotropic transport properties depending on the crystallographic direction, and epitaxial thin films enable the evaluation of anisotropic properties for different orientations and the implementation of strain for enhancing oxygen migration. Moreover, epitaxial thin films can be versatilely applied to real applications, in contrast to single crystals.

An additional comment is that higher-order RP series oxides (A_n+1_B_n_O_3n+1_ with *n* ≥ 2) seem good candidates, as well, for the enhancement of oxygen transport properties. Although oxygen ion migration in higher-order RP oxides is not fully understood, these RP oxides exhibit higher electronic conductivity and thermal stability than the *n* = 1 RP oxides. Thus, exploring new RP systems will bring new opportunities for developing materials with fast oxygen migration.

## Figures and Tables

**Table 1:** Oxygen ion migration energies in the *a-b* plane of various A_2_BO_4+δ_.

Material	E_a_/eV	Methodology	Mechanism	Ref.
La_2_NiO_4+δ_	0.29	MD	Interstitialcy mechanism	[55]
La_2_NiO_4+δ_	0.55	Static atomistic simulation	Vacancy mechanism	[66]
La_2_NiO_4+δ_	1.2	DFT	Interstitial mechanism	[67]
La_2_NiO_4+δ_	0.51	MD	Interstitialcy mechanism	[63]
Pr_2_NiO_4+δ_	0.49–0.64	MD	Interstitialcy mechanism	[68]
La_2_CoO_4+δ_	0.31	MD	Interstitialcy mechanism	[70]
La_2_CoO_4+δ_	0.73–0.80	DFT	Interstitialcy mechanism	[70]
La_2_CoO_4+δ_	1.27–1.39	DFT	Interstitial mechanism	[70]

**Table 2:** Oxygen stoichiometry in La_2−x_Sr_x_MO_4±δ_ (M = Cu, Ni, and Co) with various Sr contents. Oxygen nonstoichiometry of each of the samples was ascertained under reduction in air between 850 and 950 °C.

x (Sr)	La_2−x_Sr_x_CuO_4±δ_	La_2−x_Sr_x_NiO_4±δ_	La_2−x_Sr_x_CoO_4±δ_
	4 ± δ [Ref.]	4 ± δ [Ref.]	4 ± δ [Ref.]
0.0	4.01 [75]	4.14 [76]	4.15 [77]
0.1	4.00 [75]	4.12 [76]	−
0.2	3.99 [75]	4.11 [76]	−
0.3	3.98 [75]	4.09 [76]	−
0.5	3.90 [78]	4.06 [76]	4.07 [46]
1.0	3.60 [78]	3.99 [76]	4.00 [46]
1.4	−	3.95 [76]	3.90 [46]

**Table 3:** Activation energies of oxygen self-diffusion in A_2_BO_4+δ_.

Material	Ea/eV	Temperature Range/°C	Comment	Ref.
Pr_2_NiO_4+δ_	0.67	450–700	Single crystal, *a-b* plane	[49]
Pr_2_NiO_4+δ_	1.10	450–700	Single crystal, *c*-direction	[49]
Pr_2_NiO_4+δ_	0.76	550–850	Polycrystalline	[51]
La_2_NiO_4+δ_	0.81	525–600	Single crystal, *a-b* plane	[97]
La_2_NiO_4+δ_	0.75	450–600	Single crystal, *c*-direction	[97]
La_2_NiO_4+δ_	0.87	500–850	Polycrystalline	[51]
Nd_2_NiO_4+δ_	1.38	450–700	Single crystal, *a-b* plane	[49]
Nd_2_NiO_4+δ_	1.27	450–700	Single crystal, *c*-direction	[49]
Nd_2_NiO_4+δ_	1.05	550–850	Polycrystalline	[51]
La_2_CuO_4+δ_	0.81	390–600	Single crystal	[56]
La_2_CuO_4+δ_	1.18	527–727	Polycrystalline	[98]
La_2_CoO_4+δ_	0.13	450–700	Polycrystalline	[77]

**Table 4:** Thermal expansion coefficients (TEC) of ABO_3−δ_ and A_2_BO_4±δ_.

Material	TEC (×10^−6^ K^−1^)	Temperature/°C	Ref.
La_0.8_Sr_0.2_CoO_3−δ_	19.1	30–1000	[141]
La_0.6_Sr_0.4_CoO_3−δ_	20.5	30–1000	[142]
La_0.8_Sr_0.2_Co_0.2_Fe_0.8_O_3−δ_	15.4	100– 800	[143]
La_0.6_Sr_0.4_Co_0.2_Fe_0.8_O_3−δ_	15.3	100–600	[143]
La_2_NiO_4+δ_	11.0	650–950	[138]
La_1.8_Sr_0.2_NiO_4+δ_	11.2	650–950	[138]
La_1.6_Sr_0.4_NiO_4+δ_	12.0	650–950	[138]
La_0.6_Sr_1.4_MnO_4±δ_	13.5	30–800	[144]
La_0.2_Sr_1.8_MnO_4±δ_	16.5	30–800	[144]
La_0.5_Sr_0.5_Co_0.5_Fe_0.5_O_4−δ_	13.5	30–700	[145]
La_2_Ni_0.9_Co0.1O_4+δ_	13.8	100–900	[139]
La_1.3_Sr_0.7_CoO_4−δ_	9.6	30–1000	[27]
La_1.4_Sr_0.6_CoO_4−δ_	10.1	30–700	[145]
LaSrCoO_4−δ_	14.3	30–1000	[27]
